# Public Awareness, Attitudes, and Practices Toward Digital Health Surveillance in Malawi: Cross-Sectional Study

**DOI:** 10.2196/91033

**Published:** 2026-10-22

**Authors:** Patricia Kamanga, Jeremot Masoambeta, Vincent Kamforzi, Bertha Banda Kayuni, Isaach Ndemera, Chriswell Nkoloma, Lusungu Chisesa, Atusaye Mwalwanda, Selemani Ngwira, Hope Kalichero, Alex Thawani, Robina Chirwa Vokhiwa, Dzinkambani Kambalame, Hamdan Saidi, Peter Salilika, Tsung-Shu Joseph Wu

**Affiliations:** 1 Research Department, Luke International, P.O. Box 1088, Mzuzu, Malawi; 2 Department of Agriculture and Economics, National Statistical Office, Zomba, Malawi; 3 Department of Research, Public Health Institute of Malawi, Lilongwe, Malawi; 4 Overseas Mission Department, Pingtung Christian Hospital, No.60 Ta-Lian RoadPingtung, 90059, Taiwan, 265 999210386

**Keywords:** digital surveillance, public health, awareness, attitudes, pandemic preparedness, COVID-19, cholera

## Abstract

**Background:**

Digital surveillance systems are increasingly recognized as essential tools for enhancing early outbreak detection, reporting, and response, especially in low-resource settings. The COVID-19 pandemic unveiled the critical role of digital health surveillance systems in timely detection, monitoring, and responses to public health threats. However, successful implementation depends on public awareness, acceptance, and willingness to participate. Limited evidence exists on how the public perceives and interacts with digital surveillance in Malawi.

**Objective:**

The aim of the study was to assess the awareness, attitudes, and practices of adult Malawians toward digital surveillance during pandemics and epidemics.

**Methods:**

A cross-sectional quantitative study design was used. Data were collected in 10 selected districts (Chitipa, Mzimba South, Kasungu, Lilongwe, Salima, Balaka, Thyolo, Phalombe, Blantyre, and Chikwawa) of Malawi that had high and low levels of cholera and COVID-19 disease burden. The survey recruited 1174 participants who were 18 years or older at the time of data collection. Descriptive statistics were computed using R statistical software. Ethical approval was obtained from the National Health Sciences Research Committee prior to data collection.

**Results:**

A total of 1174 participants were surveyed, 65% (n=768) were female, and the mean age was 36.9 (SD 14.26) years. Only 59% (n=697) demonstrated awareness of digital surveillance systems, though 51% (n=603) had smartphones and 41% (n=477) used mobile phones. A positive attitude was observed in 89% (n=1044) of respondents, with most believing digital reporting could improve outbreak control, and 80% (n=939) were confident in privacy and data security. Practices related to digital reporting were low, with only 18% (n=211) having ever reported health symptoms through any digital platform.

**Conclusions:**

Enhancing digital health surveillance literacy, promoting inclusivity, and ensuring accountability will improve readiness for future pandemics through digital surveillance systems. Hence, there is a need for nationwide community sensitization programs to raise awareness about the benefits of digital health surveillance.

## Introduction

Digital surveillance, defined as the use of digital tools and platforms for real-time disease detection, reporting, and response, has become increasingly important to global health systems. In 2019, the world faced a deadly COVID-19 pandemic. The disease claimed approximately 4 million lives worldwide [1-3]. The rapid spread of the disease necessitated that surveillance activities adapt quickly to provide decision-makers with real-time data for decision-making. During the COVID-19 pandemic, various digital tools were used for public health surveillance. A total of 90 countries reported having used at least one digital technology for public health surveillance [4]. The COVID-19 pandemic highlighted the importance of timely surveillance systems capable of monitoring outbreaks and enabling rapid public health action [4]. Low- and middle-income countries, such as Malawi, stand to benefit significantly from digital surveillance due to persistent constraints in traditional surveillance systems, including delayed reporting, low coverage, and limited capacity.

With the rapid expansion of mobile penetration, digital health tools have emerged as feasible, scalable solutions. Malawi has seen notable increases in mobile ownership, use of mobile money, and expansion of digital health programs. However, technology adoption is influenced by public awareness, trust, and willingness to engage. For instance, 1 study found that the mobile contact tracing app (MCTA) was the most significant predictor of an individual’s willingness to adopt such technologies/tools and resistance to their use. Moreover, the study found that barriers to taking action positively influenced resistance, whereas personal norms negatively influenced resistance to the use of MCTA [5].

Beyond awareness and attitude, public health specialists and policymakers need to understand the levels of acceptability. Donelle et al [6] noted several factors attributing to low acceptability of digital health surveillance, deterring individuals from using technologies, including issues with limited mobile phone battery life, limited access to technology including smartphones and inadequate internet connection, users’ concerns around data privacy and security, and lack of trust.

In a multicountry study, Altmann et al [7] looked at the acceptability of app-based contact tracing for COVID-19. The study aimed to investigate the user acceptability of a contact tracing app (CTA) in 5 countries hit by the pandemic: France, Germany, Italy, the United Kingdom, and the United States. The study measured intentions to use CTAs across 2 different installation regimes (voluntary vs automatic installation by mobile phone providers). The findings showed that the app was widely supported under both regimes. However, issues of cybersecurity and privacy and a lack of trust in the government and other relevant officials were the main barriers to adopting CTAs.

Zetterholm et al [8], in their study on public acceptance of digital CTAs during COVID-19, found that, as much as acceptance varied from country to country, what was common and key was that there was lower acceptance of the CTAs among mistrusting people. This is consistent with a study on public attitudes toward digital health surveillance by Nurgalieva et al [9]. Additionally, there were disparities among people who were socially disadvantaged and people with low technical skills in lower acceptance of the CTAs.

Wnuk et al [10] conducted a study on the acceptance of COVID-19 tracking technologies: the role of perceived threats, lack of control, and ideological beliefs. The study aimed to examine if the perceived threat and lack of control during a pandemic, as well as stable ideological views, predicted the acceptance of surveillance technologies. The study’s results also showed that, despite having a smaller predictive value, there was a significant positive relationship between the perceived threat and lack of control and the acceptance of surveillance technologies.

Furthermore, during a participatory survey to evaluate the functionality and acceptability of the classical swine fever surveillance system in wild boars in Germany, the study highlighted the importance of considering actors who provide data on system acceptability as a key attribute when selecting surveillance strategies to ensure effective field implementation and continuous system improvement [11]. Digital technologies support real-time data collection, analysis, presentation, and dissemination [8,12,13], thereby enabling timely decision-making during disease outbreaks. Digital surveillance tools are therefore essential to ensure efficient data flow across all levels of the public health response.

Digital surveillance solutions in Malawi are implemented mainly using the One Health Surveillance Platform (OHSP) and District Health Information System (DHIS2). In 2022, the ministry deployed a WhatsApp COVID-19 chatbot to the general public to increase access to health services related to COVID-19. The health services that were available on the chatbot were COVID-19 surveillance, including self-reporting, as well as providing COVID-19 vaccination information and reminders for the second dose depending on the type of vaccine. Digital health surveillance has proven to be effective in infectious disease, epidemic, and pandemic prevention and control despite being new in the public health field. Several global studies have shown gaps in public awareness, attitudes, and acceptability toward digital health surveillance, and Malawi is no exception.

Despite the potential benefits, limited research has been conducted in Malawi to understand public perceptions and behaviors toward digital surveillance. Understanding community awareness, attitudes, and practices (AAPs) is critical to designing systems that are accepted, trusted, and culturally appropriate. This study, therefore, examined the AAPs of Malawians regarding digital surveillance for infectious disease detection and reporting.

## Methods

### Design

This study used a cross-sectional survey design and was conducted in 10 districts of Malawi.

### Population and Setting

The study included adults aged 18 years and older residing in sampled households within the selected enumeration areas (EAs; Table 1). Eligible participants included permanent residents able to speak the study’s supported languages (English, Chichewa, or Tumbuka). Nonresidential individuals were excluded.

**Table 1.** Sample distribution.

Region and district		EAs^a^ per district, n	Households per EA, n
Northern			
Chitipa		12	11
Mzimba South		12	11
Central			
Kasungu		12	11
Salima		12	11
Lilongwe		12	11
Southern			
Balaka		12	11
Blantyre		12	11
Chikwawa		12	11
Phalombe		12	11
Thyolo		12	11

a EA: enumeration area.

### Sample Size Determination

Sample size was estimated separately for each of Malawi’s 3 administrative regions (Northern, Central, and Southern—hereafter “regions”), which together comprise the country’s districts and served as the primary sampling strata for this study. For each region, the standard formula for estimating a single population proportion was used, applying a 95% confidence level, a 5% margin of error, and an assumed design effect of 1 in the following equation:


$$n=Z^{2}p(1-p)$$

d2

where n is the sample size per region; *Z* of 1.96, corresponding to a 95% confidence level; *P* is the estimated proportion of the population with a positive attitude toward digital health surveillance (assumed to be 0.5 to maximize sample size in the absence of prior local data); and *d* is the margin of error, set at 0.05.

This yielded a minimum required sample size of 385 respondents per region. To account for an anticipated nonresponse rate of 10%, this was adjusted upward to 428 respondents per region. Across the 3 regions, this gave a combined target of 1284 respondents (428×3). For operational feasibility, this target was rounded up and evenly distributed as 1320 households—corresponding to 12 EAs per district and 11 households per EA across the 10 purposively selected districts (see the *Sampling Procedure* section and Table 1). During household listing, 1290 of these households were confirmed to contain an eligible adult (aged ≥18 y) and were invited to participate; of these, 1174 respondents completed the survey, giving a response rate of 91% (see the *Results* section).

### Sampling Procedure

A 4-stage sampling approach was used within the 3 regions of Malawi: the Northern, Central, and Southern regions.

#### Stage 1 (District Selection)

Within each region, districts were purposively selected based on combined COVID-19 and cholera disease burden (Table 1). A combined burden score of 3.66 or higher was classified as high burden and below 3.66 as low burden. A total of 10 districts were selected: 5 with high disease burden (Mzimba South, Salima, Balaka, Lilongwe, and Blantyre) and 5 with low disease burden (Phalombe, Kasungu, Chikwawa, Thyolo, and Chitipa), distributed across the 3 regions as follows: in the northern region 2 districts were selected, in the central region 3 districts were selected, and in the southern region 5 districts were selected.

#### Stage 2 (EA Selection)

Within each selected district, 12 EAs were randomly sampled using probability-proportional-to-size sampling, favoring EAs with a higher number of households, giving 120 EAs in total (12×10 districts). The sampling frame used to select EAs was the 2018 Malawi Population and Housing Census EA frame. An EA was defined as the smallest demarcated geographical statistical unit, comprising a group of adjacent households that may form part of a village, a whole village or several villages, estates, trading or mission centers, or part of an urban area; this typically comprises approximately 250 to 300 households.

#### Stage 3 (Household Selection)

Within each selected EA, a full listing of households was conducted by the research team prior to selection. From this listing, 11 households per EA were then selected using systematic random sampling, calculated as the sampling interval (total listed households in the EA ÷ 11) with a random starting point drawn between 1 and the sampling interval. This gave a target of 132 households per district (12 EAs×11 households) and 1320 households in total across the 10 districts, consistent with Table 1. During listing and selection, 1290 of these households were confirmed to have at least one eligible resident and were retained as the invited sample.

#### Stage 4 (Respondent Selection)

Within each selected household, the household head or any other eligible adult (aged ≥18 y) present at the time of the visit was interviewed. If more than one eligible adult was present in a household, the person representing the head of the house was chosen as the respondent.

### Data Collection

Data were collected electronically using a structured questionnaire deployed on Open Data Kit (ODK) and Kobo Collector and were secured using a protected system. The questionnaire collected demographic variables and AAP measures related to digital health surveillance during pandemics. Face-to-face interviews were conducted by trained research assistants using tablets and lasted approximately 20 to 30 minutes.

### Measurements

Awareness of digital health surveillance was defined as whether respondents had previously heard of, or been exposed to, any digital health surveillance tool (eg, the WhatsApp COVID-19 chatbot, SMS-based reporting, or similar platforms), independent of how well they understood its function. It was categorized by binary responses to each awareness item (yes=“1” and no=“0”), and respondents affirming more than 50% of the awareness items were classified as “aware”; those affirming 50% or fewer were classified as “not aware.” Attitude toward digital health surveillance was defined as the usefulness, trustworthiness, and willingness to engage with digital health surveillance systems. Scoring was done using a Likert scale (“strongly disagree” to “strongly agree”) and a score above 50 means positive attitude and a lower score means negative attitude. Practice toward digital health surveillance referred to the actual use of mobile phones or digital platforms by respondents to report illness and receive health information. It was binomially coded as (yes=“1” and no=“0”) and classified as good practice with an affirmative response and bad practice otherwise.

### Data Analysis

The data were analyzed in R software version 4.4.2 (R Foundation for Statistical Computing). Descriptive statistics were used to summarize demographics using frequencies and percentages. Bivariate analyses were conducted to assess the association between sociodemographic variables and variables of interest (ownership of phone, ownership of smartphone, awareness, practice, and attitudes toward digital health surveillance tools) using Fisher exact test and the chi-square test.

### Ethical Considerations

The study was conducted after getting ethical approval from the National Health Sciences Research Committee (NHSRC; protocol number 25/05/4681), and a support letter was sought from the Secretary of Health. The district councils and the District Health Management teams were shared with the support letter from the ministry and the ethical approval certificate. The local chiefs whose community members were potential study participants were informed of the pending study in their jurisdiction. The study was carried out in compliance with local and international guidelines, codes, and statutes.

Confidentiality was strictly maintained throughout the study. Personal information, such as name, residence, or identifying markers, was not collected. Results were presented in aggregated form to ensure participants’ privacy. The data were stored on a password-protected hard drive, with access restricted to the principal investigator and coinvestigators. Participation in the study was voluntary, and participants were not penalized if they chose not to participate. Informed consent was obtained before participation, with participants either signing or placing their thumbprint on the consent form.

Participants were not paid for participating in the study. However, MWK 10,000 (at the exchange rate of MWK 1=US $0.00058 as of October 12, 2026) was given as compensation for derailing them from their responsibilities.

## Results

A total of 1290 eligible participants were invited to participate in the study. Of these 1174 responded to the questions, resulting in a response rate of 91%.

### Participant Characteristics

A total of 1174 participants were enrolled in the study. The majority were female (n=768, 65%) participants with a mean age of 36.9 (SD 14.26) years. Most respondents had attained primary education. Over half (n=661, 56%) of respondents lived in rural areas and were evenly distributed across 10 districts. Phone ownership was high (n=922, 79%), with 51% (n=603) owning smartphones. Awareness of digital health surveillance tools was reported by 59% (n=697), even though half of the respondents indicated that they were unfamiliar with digital health tools, while 74.7% (n=877) had received health information via phone, and 58% (n=683) had heard of campaigns promoting digital reporting. Only 41% (n=487) had used a phone or app to report or receive health information. However, out of the total respondents, 76% (n=889) never reported illness using electronic tools specifically. SMS (n=464, 40%) and phone calls (n=375, 32%) were the most preferred reporting methods, and 92% (n=1077) believed digital health surveillance works well. Despite positive perceptions, only 18% (n=211) had reported health information digitally in the past year (Table S3 in Multimedia Appendix 1).

### Awareness of Digital Health Surveillance

The study revealed that 59% (n=697) of respondents were aware of digital health surveillance systems (Table 2). Awareness differed significantly by education and district (*P*<.001). Regarding education, 44% (309/697) had primary education and 5% (35/697) had no formal education; regarding district of residence, in Balaka, Salima, and Chitipa, around 14% were aware of digital surveillance systems, while in Chikwawa, 5% were aware of digital surveillance systems.

**Table 2.** Awareness of digital health surveillance taken from the Pandemic Preparedness Toolkit (PPT) digital acceptability survey.

Characteristic	No (n=477)		Yes (n=697)		*P* value^a^
	n (%)	95% CI (%)	n (%)	95% CI (%)	
Sex					.14
Female	324 (68)	63-72	444 (64)	60-67	
Male	153 (32)	28-37	253 (36)	33-40	
Age group (y)					.09
18‐39	297 (62)	58-67	451 (65)	61-68	
40‐59	124 (26)	22-30	191 (27)	24-31	
60+	56 (12)	9.1-15	55 (7.9)	6.0-10	
Education					<.001
No formal education	40 (8.4)	6.1-11	35 (5)	3.6-7.0	
Primary	245 (51)	47-56	309 (44)	41-48	
Secondary	167 (35)	31-40	285 (41)	37-45	
Tertiary	25 (5.2)	3.5-7.7	68 (9.8)	7.7-12	
Location					.90
Rural	269 (56)	52-61	392 (56)	52-60	
Urban	208 (44)	39-48	305 (44)	40-48	
District					<.001
Balaka	21 (4.4)	2.8-6.8	98 (14)	12-17	
Blantyre	73 (15)	12-19	59 (8.5)	6.6-11	
Chikwawa	59 (12)	9.6-16	35 (5)	3.6-7.0	
Chitipa	31 (6.5)	4.5-9.2	101 (14)	12-17	
Kasungu	58 (12)	9.4-16	50 (7.2)	5.4-9.4	
Lilongwe	18 (3.8)	2.3-6.0	78 (11)	9.0-14	
Mzimba South	83 (17)	14-21	37 (5.3)	3.8-7.3	
Phalombe	29 (6.1)	4.2-8.7	79 (11)	9.1-14	
Salima	34 (7.1)	5.1-9.9	99 (14)	12-17	
Thyolo	71 (15)	12-18	61 (8.8)	6.8-11	

a Pearson chi-square test.

### Sources of Digital Health Surveillance Information

The main sources through which respondents accessed digital health surveillance information were the Ministry of Health (MoH) through SMS, with 59% (n=687) among female participants (*P*=.006; Table S4 in Multimedia Appendix 1). The sources and channels of use for receiving health information were commonly SMS from MoH (34.4%; Figure 1). Other sources, such as health workers, social media, friends and family, and community meetings, were reported less frequently.

**Figure 1.**
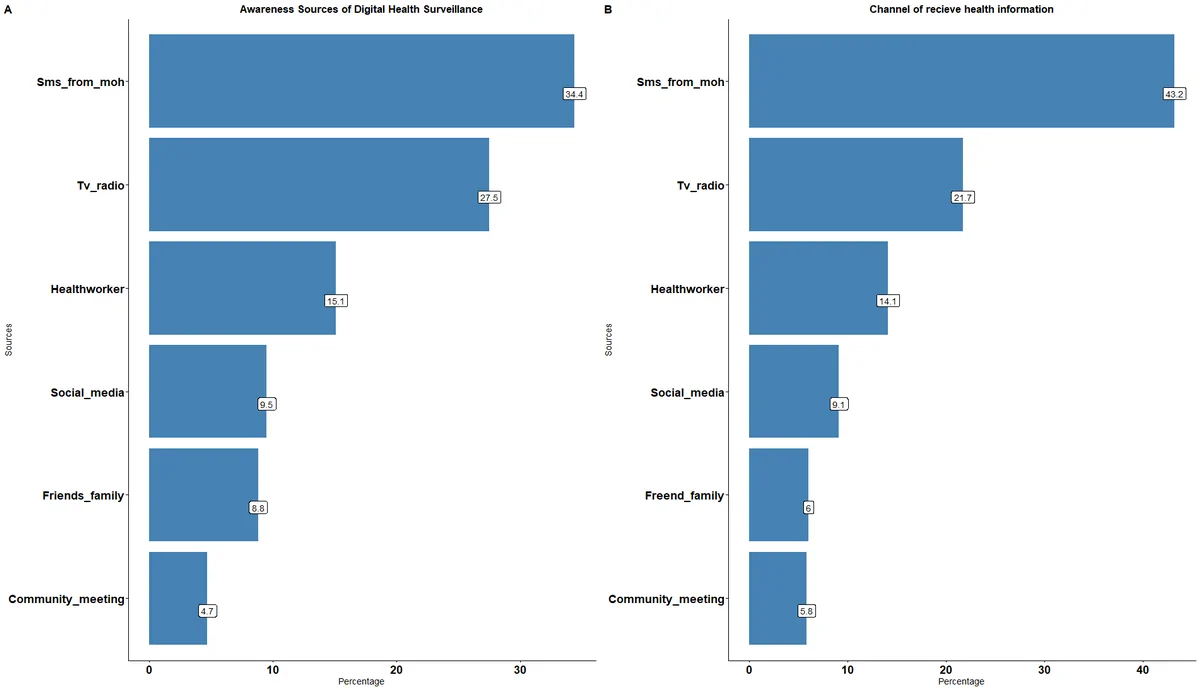
Source and channel used for receiving information on digital health. (A) Awareness sources of digital health surveillance and (B) channel of receiving health information. PPT: Pandemic Preparedness Toolkit. Source: PPT digital acceptability survey.

### Receive Health Information via Phone During the COVID-19 Pandemic or the 2022-2024 Cholera Outbreak in Malawi

The majority of participants (n=877, 75%) received COVID-19 and cholera information via phone. Receipt of information differed significantly by sex, age, education, and district (*P*<.05). By sex, female participants (64%) received information via phone. By age group, 66% (18‐39 y) received health information via phone. On education, those with primary education (43%) had received the information. Regarding the districts of Salima and Thyolo, 12% each received the information via phone (Table S5 in Multimedia Appendix 1).

### Outbreak Digital Reporting Awareness Campaigns

Out of the total sample of 1174 respondents, 683 reported awareness of digital reporting of outbreaks. Awareness was associated with the level of education (*P*=.03). Respondents with primary education (n=310, 45%) were aware of digital reporting. The district of residence was also associated with awareness (*P*<.001). Awareness was higher in Salima, Balaka, Chitipa, Kasungu, and Lilongwe. On the other hand, awareness was lower in Blantyre, Chikwawa, and Thyolo (Table S6 in Multimedia Appendix 1).

### Attitude Toward Digital Health Surveillance Tools

Most respondents (n=1044, 89%) expressed willingness to accept a digital system if approved by the MoH, and 84% agreed that using phones to report illness could improve community health. Confidence in data security was also high (80%). About 83% felt comfortable reporting symptoms through SMS or apps, while 79% agreed that people could accept digital tools once properly explained (Figure 2).

**Figure 2.**
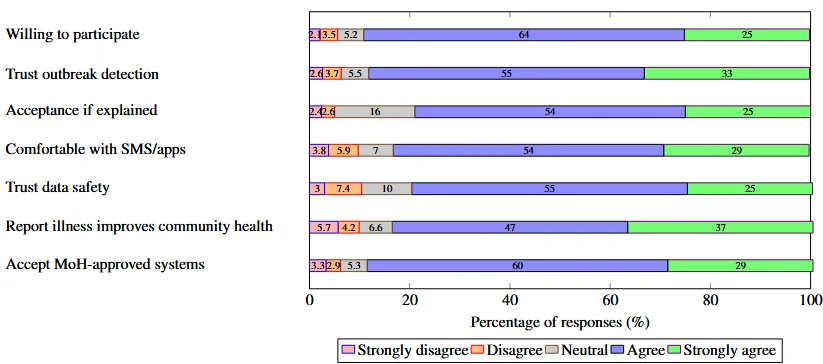
Attitudes toward digital health surveillance tools. MoH: Ministry of Health; PPT: Pandemic Preparedness Toolkit. Source: PPT digital acceptability survey.

### Belief and Comfort in Phone-Based Illness Reporting

More than half (n=232, 54%) of rural residents and 46% (n=197) of urban residents strongly agreed that using phones to report illness can improve health care in their communities (*P*=.04). Across districts, Thyolo (15%) recorded the highest level of strong agreement, while Mzimba South (6%) recorded the lowest (Table S7 in Multimedia Appendix 1). Regarding comfort in reporting health symptoms via SMS or mobile apps, participants residing in Chitipa (16%) recorded the highest level of comfort, while Lilongwe recorded the lowest (5%; *P*<.05; Table S8 in Multimedia Appendix 1).

### Public Practices of Using Digital Health Surveillance Tools

#### Use of Phone or Mobile App to Receive and Report Health Information

A significantly higher proportion of female (n=297, 61%) than male (n=190, 39%) reported using mobile phones or apps for health purposes (*P*=.007). Younger respondents (18‐39 y) showed the highest usage (n=315, 65%), while usage dropped sharply among those aged 60 years or older (n=30, 6.2%; *P*=.003). Digital health tool use increased with education. Only 3.5% (n=17) of respondents with no formal education used them, compared to 11% (n=53) among those with tertiary education (*P*<.001). Significant variation was observed across districts (*P*=.001). For example, Chitipa (n=72, 15%) had the highest usage, while Chikwawa (n=27, 5.5%) had the lowest (Table S9 in Multimedia Appendix 1).

#### Methods Preferred for Reporting Symptoms or Receiving Alerts

The preferred method of reporting was through SMS. By age, SMS preference was highest among respondents aged 18 to 39 years (n=320, 69%) and lowest among those aged 60 years and older (n=22, 4.7%) with *P*<.001. By location, the highest preference was among those in rural areas (n=259, 56%) compared with urban areas (n=205, 44%) with *P*<.001. At the district level, Salima (n=80, 17%) recorded the highest SMS reporting preference, while Kasungu (n=27, 5.8%) had the lowest (*P*<.001; Table S10 in Multimedia Appendix 1).

### Digital Health Tools Used in the Past 12 Months for Reporting Health-Related Information

By sex, out of the respondents who used the digital health tools, 57% (n=119) were female while 43% (n=89) were male (*P*=.06). By location, for those who had used the digital tools, 64% (n=134) were from rural areas while 36% (n=74) were from urban areas (*P*=.009). Another statistical significance was observed at the district level, where, out of those who used, the highest were from Balaka (n=35, 17%) and the lowest from Chikwawa (n=8, 3.8%; *P*<.001; Tables 2 and 3).

**Table 3.** Use digital health tools to report health-related information in the last 12 months. Source: Pandemic Preparedness Toolkit (PPT) acceptability survey.

Variable	No (n=966)		Yes (n=208)		*P* value^a^
	n (%)	95% CI (%)	n (%)	95% CI (%)	
Sex					.006
Female	649 (67)	64-70	119 (57)	50-64	
Male	317 (33)	30-36	89 (43)	36-50	
Age group (y)					.06
18‐39	625 (65)	62-68	123 (59)	52-66	
40‐59	246 (25)	23-28	69 (33)	27-40	
60+	95 (9.8)	8.1-12	16 (7.7)	4.6-12	
Education level					.30
No formal education	65 (6.7)	5.3-8.5	10 (4.8)	2.5-8.9	
Primary	459 (48)	44-51	95 (46)	39-53	
Secondary	371 (38)	35-42	81 (39)	32-46	
Tertiary	71 (7.3)	5.8-9.2	22 (11)	6.9-16	
Location					.009
Rural	527 (55)	51-58	134 (64)	57-71	
Urban	439 (45)	42-49	74 (36)	29-43	
District					<.001
Balaka	84 (8.7)	7.0-11	35 (17)	12-23	
Blantyre	121 (13)	11-15	11 (5.3)	2.8-9.5	
Chikwawa	86 (8.9)	7.2-11	8 (3.8)	1.8-7.7	
Chitipa	103 (11)	8.8-13	29 (14)	9.7-20	
Kasungu	87 (9.0)	7.3-11	21 (10)	6.5-15	
Lilongwe	80 (8.3)	6.7-10	16 (7.7)	4.6-12	
Mzimba South	109 (11)	9.4-13	11 (5.3)	2.8-9.5	
Phalombe	80 (8.3)	6.7-10	28 (13)	9.3-19	
Salima	102 (11)	8.7-13	31 (15)	10-21	
Thyolo	114 (12)	9.9-14	18 (8.7)	5.4-14	

a Pearson chi-square test.

### Frequency of Phone Use for Health Services

In general, the frequency was low with 691 participants reported never using their phones for health services. Significant observations were noted in use by sex, location, and district. Among those who reported weekly use, 61% (n=40) were female and 39% (n=26) were male (*P*=.003). By location, among those who reported weekly, 55% (n=36) were from rural areas and 45% (n=30) from urban areas (*P*=.045). Finally, by district of residence, the highest was in Balaka (n=14, 21%; *P*<.001) and the lowest was in Lilongwe (n=2, 3%; *P*<.001; Table S11 in Multimedia Appendix 1)

## Discussion

### Principal Findings

This study set out to assess public AAPs toward digital health surveillance among adults in 10 Malawian districts selected for contrasting COVID-19 and cholera disease burdens. Three findings stand out. First, basic mobile phone ownership was high (n=922, 79%) even though smartphone ownership was more modest (n=603, 51%), suggesting that SMS-based and feature-phone–compatible surveillance tools are likely to reach more of the population than smartphone-dependent applications in the near term. Second, awareness (n=697, 59%) and attitudes (n=1044, 89% positive) toward digital health surveillance were considerably more favorable than actual practice, with only 18% (n=208) of respondents having ever used a digital platform to report a health symptom—a pattern consistent with the well-documented “intention-behavior gap” seen in digital health adoption elsewhere. Third, confidence in data privacy and security was notably high (n=939, 80%)—a result that runs counter to much of the literature on digital surveillance acceptance cited in the *Introduction* section. Together, these findings suggest that the primary barrier to digital health surveillance uptake in this setting is not attitudinal resistance but a combination of limited exposure/awareness and limited translation of positive attitudes into actual use.

The survey found high levels of basic phone ownership compared to lower smartphone ownership, indicating widespread and increased access to digital devices, including phones, in Malawi, aligning with regional estimates showing extensive mobile phone penetration across sub-Saharan Africa, though smartphone adoption remains uneven due to cost and infrastructure barriers [14]. Evidence from other African contexts also shows that SMS-based health interventions remain effective where smartphone penetration is limited [15,16]. Therefore, Malawi’s high basic phone ownership presents a strong foundation for digital health surveillance using simple platforms like SMS.

Awareness of digital health surveillance in this study was moderately high and varied across levels of education and district of residence among the study participants. High awareness among secondary education participants is consistent with previous studies showing that educational attainment is a strong predictor of digital health literacy and adoption [17]. On the other hand, awareness differences across districts also suggest the influence of geographical inequalities, including digital infrastructure, network coverage, and exposure to health campaigns. Similar disparities have been documented in other low-resource settings, where rural populations often have limited digital exposure [18]. The preference for SMS communication from the MoH reinforces the credibility of official health communication channels, consistent with findings that trusted sources enhance uptake of health technologies [19].

This study revealed moderate familiarity with digital health surveillance, particularly among older and less educated participants. This is a result of the user interface, which requires orientation and background awareness. A previous study attributed the actual use of digital tools to familiarity, as individuals who are more confident with technology are more likely to engage with digital health programs [20].

In the current study, the lower usage of phones to report illness in the last 12 months and moderately low use of mobile phones or apps to receive health-related information or report are consistent with research indicating that while digital readiness may exist, actual behaviors often lag due to a lack of awareness and digital skills, as well as technological barriers and trust concerns [21]. Practice was associated with sex, age, education, and district of residence.

There were positive attitudes toward the use of digital health tools for public surveillance in this study, and confidence in data privacy and security was particularly high (80%). This finding is notable because it diverges from much of the international literature reviewed in the *Introduction* section, where concerns about data privacy, cybersecurity, and government trust were consistently reported as the primary barriers to accepting digital contact-tracing and surveillance tools [6-10]. Similarly, high acceptance has been reported with digital health platforms perceived as trustworthy [22], suggesting that trust itself, once present, is a strong enabler. Several explanations may account for this divergence. First, the wording of the survey item asked about general confidence in privacy and data security rather than probing specific concerns (eg, government access to data, third-party data sharing, or reidentification risk); more specific questions may have revealed lower confidence, and social desirability bias may also have inflated affirmative responses given that data were collected by research assistants face-to-face rather than anonymously. Second, this may reflect a genuine contextual difference: unlike the high-income settings where much of the comparator literature originates [6,7,12,13], most respondents in this study had limited prior exposure to digital surveillance tools (only 59% were aware of them), and lower familiarity with a technology’s actual data flows may translate into fewer specific privacy concerns rather than genuinely stronger trust. Third, high baseline trust in the MoH as a source of health information in this sample, reflected in the preference for SMS communication from the MoH noted above, may extend to trust in how MoH-linked platforms would handle personal data. Distinguishing between these explanations—genuine contextual trust, limited familiarity, or measurement artifact—would be a valuable direction for future qualitative or mixed methods research in this setting [22].

Comparing these findings with the wider literature highlights both convergence and divergence with other settings. Consistent with studies from France [1] and a multicountry comparison including developing-nation contexts [5], positive attitudes toward digital surveillance tools were common in this study despite low actual usage—suggesting that the attitude-practice gap identified here is not unique to Malawi but a recurring pattern across settings with differing income levels and digital infrastructure. However, this study’s high reported trust in data privacy and security contrasts with the consistently trust-limited findings from France, Germany, Italy, the United Kingdom, the United States [7], Poland [10], and cross-national comparisons [9], where privacy and government-trust concerns were the dominant barriers to acceptance. This divergence may reflect structural differences between Malawi and these comparator settings: lower prior exposure to large-scale digital surveillance programs (and, correspondingly, less public debate about their risks), a still-developing formal data protection regulatory environment, and a health system in which the MoH remains a comparatively high-trust institution relative to the government generally. Where similar AAP-type surveys have been conducted in other African or low-income and middle-income country settings, findings on awareness and practice gaps broadly align with this study [5,6], reinforcing that limited exposure—rather than resistance—is likely the primary barrier to digital health surveillance uptake across comparable settings, though direct like-for-like comparisons remain scarce and represent a gap in the literature that this study helps to address.

### Limitations

This study has several limitations. First, data were self-reported, which may introduce recall bias, particularly for questions asking respondents to recall past exposure to health information or campaigns. Second, selecting one eligible adult per sampled household may have introduced selection bias if household composition (eg, presence of a working-age adult during daytime visits) systematically differed by district or region. Third, the cross-sectional design limits the ability to establish causal or temporal relationships between sociodemographic characteristics and awareness, attitudes, or practices; associations reported here should be interpreted as descriptive rather than explanatory. Fourth, the 10 study districts were purposively selected based on disease burden rather than randomly sampled from all districts nationally, which limits the generalizability of these findings to Malawi as a whole; districts not represented in this sample may differ in ways that affect AAPs. Fifth, the sample size calculation assumed a design effect of 1 (ie, no clustering effect) in the absence of a locally derived estimate; because the sampling design was in fact multistage and clustered by EA, this assumption likely understates the true variance of the estimates, and CIs reported in this study should be interpreted with some caution. Finally, the survey item on confidence in data privacy and security asked a general question rather than probing specific concerns, and face-to-face data collection may have introduced social desirability bias on this and other sensitive items.

### Implications

These findings carry several implications for the design and rollout of digital health surveillance in Malawi. First, on the district-level analyses: awareness and practice varied meaningfully by district of residence, independent of the high/low disease-burden classification used for site selection. This suggests that district-level factors other than disease burden alone, such as network coverage, prior exposure to health campaigns, or local trust in health authorities, shape uptake, and that a uniform national rollout strategy may be less effective than one that allows district health offices some flexibility to adapt communication and channel choice (eg, SMS vs community radio vs in-person outreach) to local conditions. Further research directly modeling which district-level factors predict awareness and practice would help translate this into specific, targeted measures. Second, on appropriateness given current phone ownership: with 79% basic phone ownership but only 51% smartphone ownership, and just 41% having ever used a phone or app for health reporting or information, digital health surveillance strategies in Malawi should be designed around basic-phone-compatible channels (SMS, USSD [Unstructured Supplementary Service Data], and voice) as the primary access route, with smartphone-based apps treated as a supplementary rather than primary channel for the foreseeable future. Population groups most at risk of exclusion under a smartphone-first approach include older adults, those with lower educational attainment, and residents of rural or low-coverage districts, all groups in which this study found lower awareness. Mitigation strategies could include maintaining nondigital reporting channels in parallel (eg, community health workers and radio call-in lines) during any transition period, subsidizing or distributing basic phones through existing community health structures, and prioritizing SMS/USSD-based tools in the districts and demographic groups identified here as having the lowest current awareness. Digital surveillance strategies must therefore remain inclusive through both basic mobile phones and smartphone-based applications, supported by targeted communication strategies for individuals with lower education levels and older adults, if these systems are to achieve equitable population coverage rather than reinforcing existing digital divides.

### Conclusions

This study explored the acceptability of digital health surveillance among the Malawian population during public health emergencies, including the COVID-19 pandemic. Public acceptability of digital health surveillance systems is not solely determined by technological availability but is deeply rooted in ethical, social, and contextual considerations. In this study, there was high awareness, acceptability, and willingness to participate in various digital health surveillance tools. However, the usage and practices of digital health surveillance tools were low. The level of participation was influenced by the levels of trust in health authorities (mainly the MoH), data protection assurances, and perceived personal and community benefits. Therefore, strengthening digital health literacy, ensuring clear policies on data privacy, and demonstrating tangible public benefits may improve the acceptability and use of digital health surveillance tools in Malawi, as well as strengthen Malawi’s capacity for effective, sustainable, technology-driven pandemic responses.

## Supplementary material

10.2196/91033 Multimedia Appendix 1 Supplementary tables of study findings.

## Abbreviations

AAP: awareness, attitudes, and practices
CTA: contact tracing app
DHIS2: District Health Information System
EA: enumeration area
MCTA: mobile contact tracing app
MoH: Ministry of Health
NHSRC: National Health Sciences Research Committee
ODK: Open Data Kit
OHSP: One Health Surveillance Platform
USSD: Unstructured Supplementary Service Data
