# Supplementary material for: Public Awareness, Attitudes, and Practices Toward Digital Health Surveillance in Malawi: Cross-Sectional Study
Source: JMIR Public Health Surveill. 2026 Oct 22;12:e91033. doi: 10.2196/91033 (PMC13690335; doi:10.2196/91033)
Supplement: Supplementary file 1 [file publichealth-v12-e91033-s001.docx]

**Supplementary materials**

**Table S1: District Profile**

| **District** | **Projected Population (2018-2025)** | **No. of Cases of COVID-19** | **AR. COVID-19** | **No. Of Cases of Cholera** | **AR Cholera** | **Combine AR of COVID-19 and Cholera** |
| --- | --- | --- | --- | --- | --- | --- |
| Blantyre | 1, 381, 065 | 24, 895 | 18.54 | 8, 935 | 6.47 | 25.01 |
| Lilongwe | 2, 993, 163 | 20, 684 | 7.34 | 12, 803 | 4.28 | 11.62 |
| Kasungu | 950, 234 | 2, 432 | 2.65 | 92 | 0.1 | 2.75 |
| Balaka | 504, 349 | 1, 014 | 2.09 | 4, 417 | 8.76 | 10.85 |
| Thyolo | 782, 442 | 1, 085 | 1.41 | 1, 229 | 1.57 | 2.98 |
| Chikwawa | 628, 282 | 919 | 1.51 | 886 | 1.41 | 2.92 |
| Mzimba South | 1, 017, 701 | 2, 437 | 9.51 | 26 | 0.03 | 9.54 |
| Chitipa | 256, 197 | 720 | 2.88 | 118 | 0.46 | 3.34 |
| Phalombe | 490, 354 | 825 | 1.75 | 243 | 0.5 | 2.25 |
| Salima | 551, 022 | 1, 909 | 6.19 | 3, 622 | 6.55 | 12.74 |

**Table S2: Sample Distribution**

| **District** | **Number of EAs per District** | **Number of Households per EA** |
| --- | --- | --- |
| Chitipa | 12 | 11 |
| Mzimba South | 12 | 11 |
| Kasungu | 12 | 11 |
| Salima | 12 | 11 |
| Lilongwe | 12 | 11 |
| Balaka | 12 | 11 |
| Blantyre | 12 | 11 |
| Chikwawa | 12 | 11 |
| Phalombe | 12 | 11 |
| Thyolo | 12 | 11 |

Source: PPT digital acceptability survey

**Table S3: Descriptive Analysis**

| **Characteristic** | **n = 1,174**^1^ |
| --- | --- |
| **Sex** |  |
| Female | 768 (65) |
| Male | 406 (35) |
| **Age (years)** | 36.9(14.3) |
| **Education level** |  |
| No formal education | 75 (6.4) |
| Primary | 554 (47.2) |
| Secondary | 452 (38.5) |
| Tertiary | 93 (7.9) |
| **Location** |  |
| Rural | 661 (56) |
| Urban | 513 (44) |
| **District** |  |
| Balaka | 119 (10.1) |
| Blantyre | 132 (11.2) |
| Chikwawa | 94 (8) |
| Chitipa | 132 (11.2) |
| Kasungu | 108 (9.2) |
| Lilongwe | 96 (8.2) |
| Mzimba south | 120 (10.2) |
| Phalombe | 108 (9.2) |
| Salima | 133 (11.3) |
| Thyolo | 132 (11.2) |
| **Ownership of the phone** |  |
| No | 252 (21) |
| Yes | 922 (79) |
| **Ownership of a smartphone** |  |
| No | 571 (49) |
| Yes | 603 (51) |
| **Aware of Digital Health Surveillance** |  |
| No | 477 (41) |
| Yes | 697 (59) |
| **Reported illness before using electronic gauges** |  |
| No | 889 (76) |
| Yes | 285 (24) |
| **Familiar with Digital Health Tools** |  |
| Not familiar | 590 (50) |
| Somewhat familiar | 363 (31) |
| Very familiar | 221 (19) |
| **Received health information via Phone** |  |
| No | 284 (24) |
| Yes | 877 (74.7) |
| Unknown | 13 (1.53) |
| **Seen or Heard Campaigns promoting Digital Reporting** |  |
| No | 491 (42) |
| Yes | 683 (58) |
| **Use a Mobile phone or App to receive or report health information** |  |
| No | 687 (59) |
| Yes | 487 (41) |
| **Preferred methods for reporting symptoms or receiving alerts** |  |
| Community health worker | 131 (11) |
| In-person | 130 (11) |
| Mobile app | 74 (6.3) |
| Phone call | 375 (32) |
| SMS | 464 (40) |
| **The community thinks digital health surveillance works well** |  |
| No | 97 (8.3) |
| Yes | 1,077 (92) |
| **In the last 12 months, reported health-related information using a phone or a digital tool** |  |
| No | 966 (82) |
| Yes | 208 (18) |
| **Frequency of using the phone to access health services or information** |  |
| Monthly | 80 (6.8) |
| Never | 691 (58.9) |
| Rarely | 337 (28.7) |
| Weekly | 66 (5.6) |
| *^1^n (%); Mean (SD)* | |

*Source: PPT digital acceptability Survey*

**Table S4: Source of Digital Health Surveillance**

| **Characteristic** | **Community meeting** n=58*^1^*  **n(%)** | **95% CI***^2^* | **Friends/Family** n=109*^1^*  **n(%)** | **95% CI***^2^* | **Health worker** n = 186*^1^*  **n(%)** | **95% CI***^2^* | **SMS from MoH** n = 425*^1^*  **n(%)** | **95% CI***^2^* | **Social media** n = 117*^1^*  **n(%)** | **95% CI***^2^* | **TV/Radio** n = 339*^1^*  **n(%)** | **95% CI***^2^* | ***P*-value***^3^* |  |
| --- | --- | --- | --- | --- | --- | --- | --- | --- | --- | --- | --- | --- | --- | --- |
| **Sex** |  |  |  |  |  |  |  |  |  |  |  |  | **0.006** |  |
| Female | 40 (69) | 55, 80 | 67 (61) | 52, 70 | 132 (71) | 64, 77 | 250 (59) | 54, 64 | 59 (50) | 41, 60 | 214(63) | 58, 68 |  |  |
| Male | 18 (31) | 20, 45 | 42 (39) | 30, 48 | 54 (29) | 23, 36 | 175 (41) | 36, 46 | 58 (50) | 40, 59 | 125(37) | 32, 42 |  |  |
| **Age group** |  |  |  |  |  |  |  |  |  |  |  |  |  |  |
| 18-39 | 37 (64) | 50, 76 | 69 (63) | 53, 72 | 121 (65) | 58, 72 | 288 (68) | 63,72 | 89 (76) | 67, 83 | 224(66) | 61, 71 |  |  |
| 40-59 | 16 (28) | 17, 41 | 30 (28) | 20, 37 | 49 (26) | 20, 33 | 110 (26) | 22, 30 | 23 (20) | 13, 28 | 89 (26) | 22, 31 |  |  |
| 60+ | 5 (8.6) | 3.2, 20 | 10(9.2) | 4.7, 17 | 16 (8.6) | 5.2, 14 | 27 (6.4) | 4.3, 9.2 | 5 (4.3) | 1.6,10 | 26(7.7) | 5.2, 11 |  |  |
| **Education** |  |  |  |  |  |  |  |  |  |  |  |  |  |  |
| No formal education | 2 (3.4) | 0.60, 13 | 4 (3.7) | 1.2, 9.7 | 8 (4.3) | 2.0, 8.6 | 10 (2.4) | 1.2, 4.4 | 0 (0) | 0.00,4 | 20(5.9) | 3.7,9.1 |  |  |
| Primary | 30 (52) | 38, 65 | 49 (45) | 36, 55 | 92 (49) | 42, 57 | 175 (41) | 36, 46 | 25 (21) | 15, 30 | 143(42) | 37, 48 |  |  |
| Secondary | 18 (31) | 20, 45 | 40 (37) | 28, 47 | 64 (34) | 28, 42 | 193 (45) | 41, 50 | 58 (50) | 40, 59 | 146(43) | 38, 49 |  |  |
| Tertiary | 8 (14) | 6.6, 26 | 16 (15) | 8.9, 23 | 22 (12) | 7.7, 18 | 47 (11) | 8.3, 15 | 34 (29) | 21, 38 | 30(8.8) | 6.1, 13 |  |  |
| **Location** |  |  |  |  |  |  |  |  |  |  |  |  | 0.10 |  |
| Rural | 39 (67) | 54, 79 | 70 (64) | 54, 73 | 106 (57) | 50, 64 | 226 (53) | 48, 58 | 58 (50) | 40, 59 | 187(55) | 50, 61 |  |  |
| Urban | 19 (33) | 21, 46 | 39 (36) | 27, 46 | 80 (43) | 36, 50 | 199 (47) | 42, 52 | 59 (50) | 41, 60 | 152(45) | 39, 50 |  |  |
| **District** |  |  |  |  |  |  |  |  |  |  |  |  |  |  |
| Balaka | 17 (29) | 18, 43 | 29 (27) | 19, 36 | 36 (19) | 14, 26 | 52 (12) | 9.3, 16 | 14 (12) | 6.9,20 | 67 (20) | 16, 24 |  |  |
| Blantyre | 1 (1.7) | 0.09, 10 | 6 (5.5) | 2.3, 12 | 8 (4.3) | 2.0, 8.6 | 34 (8.0) | 5.7, 11 | 8 (6.8) | 3.2,13 | 16(4.7) | 2.8,7.7 |  |  |
| Chikwawa | 2 (3.4) | 0.60, 13 | 7 (6.4) | 2.8, 13 | 4 (2.2) | 0.69,5.8 | 26 (6.1) | 4.1, 8.9 | 8 (6.8) | 3.2,13 | 5 (1.5) | 0.54,3.6 |  |  |
| Chitipa | 18 (31) | 20, 45 | 22 (20) | 13, 29 | 37 (20) | 15, 27 | 66 (16) | 12, 19 | 22 (19) | 12, 27 | 44 (13) | 9.7, 17 |  |  |
| Kasungu | 2 (3.4) | 0.60, 13 | 4 (3.7) | 1.2, 9.7 | 13 (7.0) | 3.9, 12 | 40 (9.4) | 6.9, 13 | 5 (4.3) | 1.6,10 | 32(9.4) | 6.6, 13 |  |  |
| Lilongwe | 3 (5.2) | 1.3, 15 | 19 (17) | 11, 26 | 14 (7.5) | 4.3, 13 | 54 (13) | 9.8, 16 | 10 (8.5) | 4.4,16 | 51 (15) | 12, 19 |  |  |
| Mzimba South | 1 (1.7) | 0.0, 10 | 1 (0.9) | 0.05, 5.7 | 10 (5.4) | 2.8, 10 | 18 (4.2) | 2.6, 6.7 | 4 (3.4) | 1.1,9 | 16(4.7) | 2.8,7.7 |  |  |
| Phalombe | 6 (10) | 4.3, 22 | 6 (5.5) | 2.3, 12 | 23 (12) | 8.2, 18 | 42 (9.9) | 7.3, 13 | 16 (14) | 8.3,22 | 27(8.0) | 5.4, 12 |  |  |
| Salima | 5 (8.6) | 3.2, 20 | 8 (7.3) | 3.5, 14 | 34 (18) | 13, 25 | 60 (14) | 11, 18 | 13 (11) | 6.3,19 | 56 (17) | 13, 21 |  |  |
| Thyolo | 3 (5.2) | 1.3, 15 | 7 (6.4) | 2.8, 13 | 7 (3.8) | 1.7, 7.9 | 33 (7.8) | 5.5, 11 | 17 (15) | 8.9,23 | 25(7.4) | 4.9, 11 |  |  |
| *^1^*n (%); *^2^*CI = Confidence Interval; *^3^*Pearson's Chi-squared test. Source: PPT digital acceptability survey | | | | | | | | | | | | | | |

**Table S5: Receive health information via phone**

| **Characteristic** | **No** n = (284*^1^***)** | | **Yes** (n = 877*^1^***)** | | ***P*-value***^3^* |
| --- | --- | --- | --- | --- | --- |
|  | **n(%)** | **95% CI***^2^* | **n(%)** | **95% CI***^2^* |  |
| **Sex** |  |  |  |  | **0.036** |
| Female | 201 (71) | 65, 76 | 561 (64) | 61, 67 |  |
| Male | 83 (29) | 24, 35 | 316 (36) | 33, 39 |  |
| **Age group** |  |  |  |  | **<0.001** |
| 18-39 | 152 (54) | 48, 59 | 583 (66) | 63, 70 |  |
| 40-59 | 78 (27) | 22, 33 | 237 (27) | 24, 30 |  |
| 60+ | 54 (19) | 15, 24 | 57 (6.5) | 5.0, 8.4 |  |
| **Education** |  |  |  |  | **<0.001** |
| No formal education | 33 (12) | 8.2, 16 | 42 (4.8) | 3.5, 6.5 |  |
| Primary | 171 (60) | 54, 66 | 378 (43) | 40, 46 |  |
| Secondary | 78 (27) | 22, 33 | 368 (42) | 39, 45 |  |
| Tertiary | 2 (0.7) | 0.12, 2.8 | 89 (10) | 8.3, 12 |  |
| **Location** |  |  |  |  | 0.13 |
| Rural | 172 (61) | 55, 66 | 486 (55) | 52, 59 |  |
| Urban | 112 (39) | 34, 45 | 391 (45) | 41, 48 |  |
| **District** |  |  |  |  | **0.018** |
| Balaka | 40 (14) | 10, 19 | 79 (9.0) | 7.2, 11 |  |
| Blantyre | 31 (11) | 7.6, 15 | 88 (10) | 8.2, 12 |  |
| Chikwawa | 27 (9.5) | 6.5, 14 | 67 (7.6) | 6.0, 9.7 |  |
| Chitipa | 27 (9.5) | 6.5, 14 | 105 (12) | 9.9, 14 |  |
| Kasungu | 35 (12) | 8.8, 17 | 73 (8.3) | 6.6, 10 |  |
| Lilongwe | 20 (7.0) | 4.5, 11 | 76 (8.7) | 6.9, 11 |  |
| Mzimba South | 32 (11) | 7.9, 16 | 88 (10) | 8.2, 12 |  |
| Phalombe | 16 (5.6) | 3.4, 9.2 | 92 (10) | 8.6, 13 |  |
| Salima | 31 (11) | 7.6, 15 | 102 (12) | 9.6, 14 |  |
| Thyolo | 25 (8.8) | 5.9, 13 | 107 (12) | 10, 15 |  |
| *^1^*n (%); *^2^*CI = Confidence Interval; *^3^*Pearson's Chi-squared test | | | | | |

*Source: Digital Acceptability study*

**Table S6: Awareness of campaigns promoting digital reporting of outbreaks**

| **Characteristic** | **No** (n = 491*^1^***)** | | **Yes (**n = 683*^1^***)** | | ***P*-value***^3^* |
| --- | --- | --- | --- | --- | --- |
|  | **n(%)** | **95% CI***^2^* | **n(%)** | **95% CI***^2^* |  |
| **Sex** |  |  |  |  | 0.3 |
| Female | 330 (67) | 63, 71 | 438 (64) | 60, 68 |  |
| Male | 161 (33) | 29, 37 | 245 (36) | 32, 40 |  |
| **Age group** |  |  |  |  | 0.3 |
| 18-39 | 301 (61) | 57, 66 | 447 (65) | 62, 69 |  |
| 40-59 | 137 (28) | 24, 32 | 178 (26) | 23, 30 |  |
| 60+ | 53 (11) | 8.3, 14 | 58 (8.5) | 6.6, 11 |  |
| **Education** |  |  |  |  | **0.030** |
| No Formal Education | 39 (7.9) | 5.8, 11 | 36 (5.3) | 3.8, 7.3 |  |
| Primary | 244 (50) | 45, 54 | 310 (45) | 42, 49 |  |
| Secondary | 178 (36) | 32, 41 | 274 (40) | 36, 44 |  |
| Tertiary | 30 (6.1) | 4.2, 8.7 | 63 (9.2) | 7.2, 12 |  |
| **Location** |  |  |  |  | 0.7 |
| Rural | 280 (57) | 53, 61 | 381 (56) | 52, 60 |  |
| Urban | 211 (43) | 39, 47 | 302 (44) | 40, 48 |  |
| **District** |  |  |  |  | **<0.001** |
| Balaka | 30 (6.1) | 4.2, 8.7 | 89 (13) | 11, 16 |  |
| Blantyre | 79 (16) | 13, 20 | 53 (7.8) | 5.9, 10 |  |
| Chikwawa | 75 (15) | 12, 19 | 19 (2.8) | 1.7, 4.4 |  |
| Chitipa | 43 (8.8) | 6.5, 12 | 89 (13) | 11, 16 |  |
| Kasungu | 32 (6.5) | 4.6, 9.2 | 76 (11) | 8.9, 14 |  |
| Lilongwe | 17 (3.5) | 2.1, 5.6 | 79 (12) | 9.3, 14 |  |
| Mzimba South | 63 (13) | 10, 16 | 57 (8.3) | 6.4, 11 |  |
| Phalombe | 45 (9.2) | 6.8, 12 | 63 (9.2) | 7.2, 12 |  |
| Salima | 31 (6.3) | 4.4, 8.9 | 102 (15) | 12, 18 |  |
| Thyolo | 76 (15) | 12, 19 | 56 (8.2) | 6.3, 11 |  |
| *^1^*n (%);*^2^*CI = Confidence Interval; *^3^*Pearson's Chi-squared test | | | | | |
| *Source: PPT digital acceptability Survey* | | | | | |

**Table S7: Belief in phone-based illness reporting**

| **Variable** | **Strongly Disagree n(%)** | **Strongly Disagree 95% CI** | **Disagree n(%)** | **Disagree 95% CI** | **Neutral n(%)** | **Neutral**  **95% CI** | **Agree n(%)** | **Agree 95% CI** | **Strongly Agree n(%)** | **Strongly Agree 95% CI** | ***P*-value** |
| --- | --- | --- | --- | --- | --- | --- | --- | --- | --- | --- | --- |
| **Sex** |  |  |  |  |  |  |  |  |  |  | 0.1286 |
| Female | 44 (63.8) | 51.2-74.7 | 39 (79.6) | 65.2-89.3 | 49(63.6) | 51.8-74.1 | 369 (67.1) | 63-71 | 267 (62.2) | 57.4-66.8 |  |
| Male | 25 (36.2) | 25.3-48.8 | 10 (20.4) | 10.7-34.8 | 28(36.4) | 25.9-48.2 | 181 (32.9) | 29-37 | 162 (37.8) | 33.2-42.6 |  |
| **Age group** |  |  |  |  |  |  |  |  |  |  | 0.5507 |
| 18-39 | 44 (63.8) | 51.2-74.7 | 35 (71.4) | 56.5-83 | 50(64.9) | 53.1-75.2 | 361 (65.6) | 61.5-69.6 | 258 (60.1) | 55.3-64.8 |  |
| 40-59 | 17 (24.6) | 15.4-36.7 | 10 (20.4) | 10.7-34.8 | 21(27.3) | 18-38.8 | 135 (24.5) | 21-28.4 | 132 (30.8) | 26.5-35.4 |  |
| 60 above | 8 (11.6) | 5.5-22.1 | 4 (8.2) | 2.6-20.5 | 6 (7.8) | 3.2-16.8 | 54 (9.8) | 7.5-12.7 | 39 (9.1) | 6.6-12.3 |  |
| **Education** |  |  |  |  |  |  |  |  |  |  | 0.0588 |
| No formal education | 7 (10.1) | 4.5-20.4 | 1 (2) | 0.1-12.2 | 7 (9.1) | 4-18.4 | 32 (5.8) | 4.1-8.2 | 28 (6.5) | 4.5-9.4 |  |
| Primary | 26 (37.7) | 26.5-50.2 | 18 (36.7) | 23.8-51.7 | 47 (61) | 49.2-71.7 | 265 (48.2) | 43.9-52.4 | 198 (46.2) | 41.4-51 |  |
| Secondary | 29 (42) | 30.4-54.5 | 25 (51) | 36.5-65.4 | 21(27.3) | 18-38.8 | 215 (39.1) | 35-43.3 | 162 (37.8) | 33.2-42.6 |  |
| Tertiary | 7 (10.1) | 4.5-20.4 | 5 (10.2) | 3.8-23 | 2 (2.6) | 0.5-9.9 | 38 (6.9) | 5-9.4 | 41 (9.6) | 7-12.8 |  |
| **Location** |  |  |  |  |  |  |  |  |  |  | **0.0407** |
| Rural | 50 (72.5) | 60.2-82.2 | 25 (51) | 36.5-65.4 | 48(62.3) | 50.5-72.9 | 306 (55.6) | 51.4-59.8 | 232 (54.1) | 49.2-58.9 |  |
| Urban | 19 (27.5) | 17.8-39.8 | 24 (49) | 34.6-63.5 | 29(37.7) | 27.1-49.5 | 244 (44.4) | 40.2-48.6 | 197 (45.9) | 41.1-50.8 |  |
| **District** |  |  |  |  |  |  |  |  |  |  | **0.0000** |
| Balaka | 3 (4.3) | 1.1-13 | 4 (8.2) | 2.6-20.5 | 7 (9.1) | 4-18.4 | 57 (10.4) | 8-13.3 | 48 (11.2) | 8.4-14.7 |  |
| Blantyre | 4 (5.8) | 1.9-14.9 | 0 (0) | 0-9.1 | 7 (9.1) | 4-18.4 | 73 (13.3) | 10.6-16.5 | 48 (11.2) | 8.4-14.7 |  |
| Chikwawa | 3 (4.3) | 1.1-13 | 5 (10.2) | 3.8-23 | 8 (10.4) | 4.9-20 | 33 (6) | 4.2-8.4 | 45 (10.5) | 7.8-13.9 |  |
| Chitipa | 4 (5.8) | 1.9-14.9 | 5 (10.2) | 3.8-23 | 12(15.6) | 8.7-26 | 57 (10.4) | 8-13.3 | 54 (12.6) | 9.7-16.2 |  |
| Kasungu | 5 (7.2) | 2.7-16.8 | 4 (8.2) | 2.6-20.5 | 7 (9.1) | 4-18.4 | 64 (11.6) | 9.1-14.7 | 28 (6.5) | 4.5-9.4 |  |
| Lilongwe | 0 (0) | 0-6.6 | 3 (6.1) | 1.6-17.9 | 0 (0) | 0-5.9 | 61 (11.1) | 8.7-14.1 | 32 (7.5) | 5.2-10.5 |  |
| Mzimba South | 12 (17.4) | 9.7-28.8 | 6 (12.2) | 5.1-25.5 | 5 (6.5) | 2.4-15.2 | 73 (13.3) | 10.6-16.5 | 24 (5.6) | 3.7-8.3 |  |
| Phalombe | 15 (21.7) | 13.1-33.6 | 8 (16.3) | 7.8-30.2 | 8 (10.4) | 4.9-20 | 39 (7.1) | 5.2-9.6 | 38 (8.9) | 6.4-12. |  |
| Salima | 2 (2.9) | 0.5-11 | 6 (12.2) | 5.1-25.5 | 15(19.5) | 11.7-30.4 | 63 (11.5) | 9-14.5 | 47 (11) | 8.2-14.4 |  |
| Thyolo | 21 (30.4) | 20.2-42.8 | 8 (16.3) | 7.8-30.2 | 8 (10.4) | 4.9-20 | 30 (5.5) | 3.8-7.8 | 65 (15.2) | 12-19 |  |

*Source: PPT Acceptability Survey*

**Table S8: Comfortable in reporting health symptoms via SMS/mobile applications**

| **Variable** | **Strongly Disagree n(%)** | **Strongly Disagree 95% CI** | **Disagree n(%)** | **Disagree 95% CI** | **Neutral n(%)** | **Neutral**  **95% CI** | **Agree n(%)** | **Agree**  **95% CI** | **Strongly Agree n(%)** | **Strongly Agree**  **95% CI** | ***P*-value** |
| --- | --- | --- | --- | --- | --- | --- | --- | --- | --- | --- | --- |
| **Sex** |  |  |  |  |  |  |  |  |  |  | 0.5732 |
| Female | 30 (66.7) | 50.9-79.6 | 47 (68.1) | 55.7-78.5 | 54 (65.9) | 54.5-75.7 | 427(66.9) | 63.1-70.5 | 210(61.8) | 56.3-66.9 |  |
| Male | 15 (33.3) | 20.4-49.1 | 22 (31.9) | 21.5-44.3 | 28 (34.1) | 24.3-45.5 | 211(33.1) | 29.5-36.9 | 130 (38.2) | 33.1-43.7 |  |
| **Age group** |  |  |  |  |  |  |  |  |  |  | 0.1864 |
| 18-39 | 30 (66.7) | 50.9-79.6 | 40 (58) | 45.5-69.6 | 59 (72) | 60.8-81 | 415(65) | 61.2-68.7 | 204 (60) | 54.6-65.2 |  |
| 40-59 | 13 (28.9) | 16.8-44.5 | 18 (26.1) | 16.6-38.3 | 17 (20.7) | 12.9-31.4 | 161(25.2) | 21.9-28.8 | 106(31.2) | 26.3-36.4 |  |
| 60 above | 2 (4.4) | 0.8-16.4 | 11 (15.9) | 8.6-27.2 | 6 (7.3) | 3-15.8 | 62 (9.7) | 7.6-12.3 | 30 (8.8) | 6.1-12.5 |  |
| **Education** |  |  |  |  |  |  |  |  |  |  | 0.2823 |
| No formal education | 5 (11.1) | 4.2-24.8 | 5 (7.2) | 2.7-16.8 | 7 (8.5) | 3.8-17.3 | 37 (5.8) | 4.2-8 | 21 (6.2) | 4-9.4 |  |
| Primary | 15 (33.3) | 20.4-49.1 | 31 (44.9) | 33.1-57.3 | 41 (50) | 39.4-60.6 | 314(49.2) | 45.3-53.2 | 153 (45) | 39.7-50.5 |  |
| Secondary | 23 (51.1) | 36-66.1 | 31 (44.9) | 33.1-57.3 | 29 (35.4) | 25.3-46.8 | 238(37.3) | 33.6-41.2 | 131 (38.5) | 33.4-44 |  |
| Tertiary | 2 (4.4) | 0.8-16.4 | 2 (2.9) | 0.5-11 | 5 (6.1) | 2.3-14.3 | 49 (7.7) | 5.8-10.1 | 35 (10.3) | 7.4-14.1 |  |
| **Location** |  |  |  |  |  |  |  |  |  |  | 0.1902 |
| Rural | 29 (64.4) | 48.7-77.7 | 47 (68.1) | 55.7-78.5 | 47 (57.3) | 45.9-68 | 355(55.6) | 51.7-59.5 | 183(53.8) | 48.-59.2 |  |
| Urban | 16 (35.6) | 22.3-51.3 | 22 (31.9) | 21.5-44.3 | 35 (42.7) | 32-54.1 | 283(44.4) | 40.5-48.3 | 157(46.2) | 40.8-51.6 |  |
| **District** |  |  |  |  |  |  |  |  |  |  | **0.0000** |
| Balaka | 2 (4.4) | 0.8-16.4 | 9 (13) | 6.5-23.8 | 11 (13.4) | 7.2-23.2 | 66 (10.3) | 8.1-13 | 31 (9.1) | 6.4-12.8 |  |
| Blantyre | 1 (2.2) | 0.1-13.2 | 3 (4.3) | 1.1-13 | 7 (8.5) | 3.8-17.3 | 72 (11.3) | 9-14.1 | 49 (14.4) | 10.9-18.7 |  |
| Chikwawa | 3 (6.7) | 1.7-19.3 | 10 (14.5) | 7.5-25.5 | 8 (9.8) | 4.6-18.8 | 43 (6.7) | 5-9 | 30 (8.8) | 6.1-12.5 |  |
| Chitipa | 1 (2.2) | 0.1-13.2 | 5 (7.2) | 2.7-16.8 | 11 (13.4) | 7.2-23.2 | 61 (9.6) | 7.4-12.2 | 54 (15.9) | 12.3-20.3 |  |
| Kasungu | 3 (6.7) | 1.7-19.3 | 8 (11.6) | 5.5-22.1 | 3 (3.7) | 0.9-11.1 | 66 (10.3) | 8.1-13 | 28 (8.2) | 5.6-11.8 |  |
| Lilongwe | 1 (2.2) | 0.1-13.2 | 6 (8.7) | 3.6-18.6 | 3 (3.7) | 0.9-11.1 | 70 (11) | 8.7-13.7 | 16 (4.7) | 2.8-7.7 |  |
| Mzimba South | 7 (15.6) | 7-30.1 | 4 (5.8) | 1.9-14.9 | 3 (3.7) | 0.9-11.1 | 84 (13.2) | 10.7-16.1 | 22 (6.5) | 4.2-9.8 |  |
| Phalombe | 12 (26.7) | 15.1-42.2 | 6 (8.7) | 3.6-18.6 | 7 (8.5) | 3.8-17.3 | 50 (7.8) | 5.9-10.3 | 33 (9.7) | 6.9-13.5 |  |
| Salima | 2 (4.4) | 0.8-16.4 | 4 (5.8) | 1.9-14.9 | 19 (23.2) | 14.9-34 | 83 (13) | 10.5-15.9 | 25 (7.4) | 4.9-10.8 |  |
| Thyolo | 13 (28.9) | 16.8-44.5 | 14 (20.3) | 11.9-32 | 10 (12.2) | 6.3-21.7 | 43 (6.7) | 5-9 | 52 (15.3) | 11.7-19.7 |  |

**Table S9: Use mobile phones or apps to receive and report health information by demographic characteristics**

| **Variable** | **No** n = 687*^1^* | **95% CI***^2^* | **Yes** n = 487*^1^* | **95% CI***^2^* | ***P*-value***^3^* |
| --- | --- | --- | --- | --- | --- |
| **Sex** |  |  |  |  | **0.007** |
| Female | 471 (69) | 65, 72 | 297 (61) | 56, 65 |  |
| Male | 216 (31) | 28, 35 | 190 (39) | 35, 44 |  |
| **Age group** |  |  |  |  | **0.003** |
| 18-39 | 433 (63) | 59, 67 | 315 (65) | 60, 69 |  |
| 40-59 | 173 (25) | 22, 29 | 142 (29) | 25, 33 |  |
| 60+ | 81 (12) | 9.5, 14 | 30 (6.2) | 4.3, 8.8 |  |
| **Education level** |  |  |  |  | **<0.001** |
| No Formal Education | 58 (8.4) | 6.5, 11 | 17 (3.5) | 2.1, 5.6 |  |
| Primary | 342 (50) | 46, 54 | 212 (44) | 39, 48 |  |
| Secondary | 247 (36) | 32, 40 | 205 (42) | 38, 47 |  |
| Tertiary | 40 (5.8) | 4.2, 7.9 | 53 (11) | 8.3, 14 |  |
| **Location** |  |  |  |  | 0.13 |
| Rural | 374 (54) | 51, 58 | 287 (59) | 54, 63 |  |
| Urban | 313 (46) | 42, 49 | 200 (41) | 37, 46 |  |
| **District** |  |  |  |  | **0.001** |
| Balaka | 65 (9.5) | 7.4, 12 | 54 (11) | 8.5, 14 |  |
| Blantyre | 84 (12) | 9.9, 15 | 48 (9.9) | 7.4, 13 |  |
| Chikwawa | 67 (9.8) | 7.7, 12 | 27 (5.5) | 3.8, 8.1 |  |
| Chitipa | 60 (8.7) | 6.8, 11 | 72 (15) | 12, 18 |  |
| Kasungu | 71 (10) | 8.2, 13 | 37 (7.6) | 5.5, 10 |  |
| Lilongwe | 55 (8.0) | 6.1, 10 | 41 (8.4) | 6.2, 11 |  |
| Mzimba South | 79 (11) | 9.3, 14 | 41 (8.4) | 6.2, 11 |  |
| Phalombe | 65 (9.5) | 7.4, 12 | 43 (8.8) | 6.5, 12 |  |
| Salima | 67 (9.8) | 7.7, 12 | 66 (14) | 11, 17 |  |
| Thyolo | 74 (11) | 8.6, 13 | 58 (12) | 9.2, 15 |  |
| *^1^*n (%); *^2^*CI = Confidence Interval; *^3^*Pearson's Chi-squared test. *Source: PPT digital acceptability Survey* | | | | | |

**Table S10: Method preferred for reporting symptoms or receiving alerts**

| **Variable** | **Community health worker** n = 131^1^ | **95% CI**^2^ | **In-person** n = 130^1^ | **95% CI**^2^ | **Mobile app** n = 74^1^ | **95% CI**^2^ | **Phone call** n = 375^1^ | **95% CI**^2^ | **SMS** n = 464^1^ | **95% CI**^2^ | ***P*-value**^3^ |
| --- | --- | --- | --- | --- | --- | --- | --- | --- | --- | --- | --- |
| **Sex** |  |  |  |  |  |  |  |  |  |  | 0.10 |
| female | 94 (72) | 63, 79 | 94 (72) | 64, 80 | 45 (61) | 49, 72 | 246 (66) | 61, 70 | 289 (62) | 58, 67 |  |
| male | 37 (28) | 21, 37 | 36 (28) | 20, 36 | 29 (39) | 28, 51 | 129 (34) | 30, 39 | 175 (38) | 33, 42 |  |
| **Age group** |  |  |  |  |  |  |  |  |  |  | **<0.001** |
| 18-39 | 66 (50) | 42, 59 | 83 (64) | 55, 72 | 52 (70) | 58, 80 | 227 (61) | 55, 65 | 320 (69) | 65, 73 |  |
| 40-59 | 44 (34) | 26, 42 | 25 (19) | 13, 27 | 18 (24) | 15, 36 | 106 (28) | 24, 33 | 122 (26) | 22, 31 |  |
| 60+ | 21 (16) | 10, 24 | 22 (17) | 11, 25 | 4 (5.4) | 1.7, 14 | 42 (11) | 8.3, 15 | 22 (4.7) | 3.1, 7.2 |  |
| **Education level** |  |  |  |  |  |  |  |  |  |  |  |
| No formal education | 17 (13) | 8.0, 20 | 16 (12) | 7.4, 20 | 2 (2.7) | 0.47, 10 | 24 (6.4) | 4.2, 9.5 | 16 (3.4) | 2.1, 5.7 |  |
|  |  |  |  |  |  |  |  |  |  |  |  |
| Primary | 66 (50) | 42, 59 | 68 (52) | 43, 61 | 10 (14) | 7.0, 24 | 198 (53) | 48, 58 | 212 (46) | 41, 50 |  |
| Secondary | 44 (34) | 26, 42 | 39 (30) | 22, 39 | 42 (57) | 45, 68 | 130 (35) | 30, 40 | 197 (42) | 38, 47 |  |
| Tertiary | 4 (3.1) | 0.98, 8.1 | 7 (5.4) | 2.4, 11 | 20 (27) | 18, 39 | 23 (6.1) | 4.0, 9.2 | 39 (8.4) | 6.1, 11 |  |
| **Location** |  |  |  |  |  |  |  |  |  |  | **<0.001** |
| rural | 91 (69) | 61, 77 | 78 (60) | 51, 68 | 27 (36) | 26, 49 | 206 (55) | 50, 60 | 259 (56) | 51, 60 |  |
| urban | 40 (31) | 23, 39 | 52 (40) | 32, 49 | 47 (64) | 51, 74 | 169 (45) | 40, 50 | 205 (44) | 40, 49 |  |
| **District** |  |  |  |  |  |  |  |  |  |  | **<0.001** |
| Balaka | 21 (16) | 10, 24 | 9 (6.9) | 3.4, 13 | 2 (2.7) | 0.47, 10 | 31 (8.3) | 5.8, 12 | 56 (12) | 9.3, 15 |  |
| Blantyre | 11 (8.4) | 4.5, 15 | 15 (12) | 6.8, 19 | 10 (14) | 7.0, 24 | 37 (9.9) | 7.1, 13 | 59 (13) | 9.9, 16 |  |
| Chikwawa | 6 (4.6) | 1.9, 10 | 19 (15) | 9.3, 22 | 1 (1.4) | 0.07, 8.3 | 33 (8.8) | 6.2, 12 | 35 (7.5) | 5.4, 10 |  |
| Chitipa | 7 (5.3) | 2.4, 11 | 14 (11) | 6.2, 18 | 4 (5.4) | 1.7, 14 | 50 (13) | 10, 17 | 57 (12) | 9.5, 16 |  |
| Kasungu | 16 (12) | 7.4, 19 | 10 (7.7) | 4.0, 14 | 9 (12) | 6.1, 22 | 46 (12) | 9.2, 16 | 27 (5.8) | 3.9, 8.5 |  |
| Lilongwe | 9 (6.9) | 3.4, 13 | 16 (12) | 7.4, 20 | 3 (4.1) | 1.1, 12 | 33 (8.8) | 6.2, 12 | 35 (7.5) | 5.4, 10 |  |
| Mzimba South | 9 (6.9) | 3.4, 13 | 11 (8.5) | 4.5, 15 | 10 (14) | 7.0, 24 | 56 (15) | 12, 19 | 34 (7.3) | 5.2, 10 |  |
| Phalombe | 23 (18) | 12, 25 | 13 (10) | 5.6, 17 | 12 (16) | 9.0, 27 | 31 (8.3) | 5.8, 12 | 29 (6.3) | 4.3, 9.0 |  |
| Salima | 10 (7.6) | 3.9, 14 | 7 (5.4) | 2.4, 11 | 9 (12) | 6.1, 22 | 27 (7.2) | 4.9, 10 | 80 (17) | 14, 21 |  |
| Thyolo | 19 (15) | 9.2, 22 | 16 (12) | 7.4, 20 | 14 (19) | 11, 30 | 31 (8.3) | 5.8, 12 | 52 (11) | 8.6, 15 |  |
| ^1^n (%);^2^CI = Confidence Interval; ^3^Pearson's Chi-squared test. *Source: PPT digital acceptability Survey* | | | | | | | | | | | |

**Table S11: Frequency of access to health services using digital tools**

| **Variable** | **Monthly** N = 80*^1^* | **95 CI***^2^* | **Never** N = 691*^1^* | **95 CI***^2^* | **Rarely** N = 337*^1^* | **95 CI***^2^* | **Weekly** N = 66*^1^* | **95 CI***^2^* | **p-value***^3^* |
| --- | --- | --- | --- | --- | --- | --- | --- | --- | --- |
| **Sex** |  |  |  |  |  |  |  |  | **0.003** |
| Female | 40 (50) | 39, 61 | 476 (69) | 65, 72 | 212 (63) | 57, 68 | 40 (61) | 48, 72 |  |
| Male | 40 (50) | 39, 61 | 215 (31) | 28, 35 | 125 (37) | 32, 43 | 26 (39) | 28, 52 |  |
| **Age group** |  |  |  |  |  |  |  |  | 0.10 |
| 18-39 | 48 (60) | 48, 71 | 434 (63) | 59, 66 | 218 (65) | 59, 70 | 48 (73) | 60, 83 |  |
| 40-59 | 29 (36) | 26, 48 | 181 (26) | 23, 30 | 90 (27) | 22, 32 | 15 (23) | 14, 35 |  |
| 60+ | 3 (3.8) | 0.97, 11 | 76 (11) | 8.8, 14 | 29 (8.6) | 5.9, 12 | 3 (4.5) | 1.2, 14 |  |
| **Education level** |  |  |  |  |  |  |  |  |  |
| No Formal education | 6 (7.5) | 3.1, 16 | 54 (7.8) | 6.0, 10 | 12 (3.6) | 1.9, 6.3 | 3 (4.5) | 1.2, 14 |  |
| Primary | 35 (44) | 33, 55 | 340 (49) | 45, 53 | 155 (46) | 41, 51 | 24 (36) | 25, 49 |  |
| Secondary | 30 (38) | 27, 49 | 255 (37) | 33, 41 | 140 (42) | 36, 47 | 27 (41) | 29, 54 |  |
| Tertiary | 9 (11) | 5.6, 21 | 42 (6.1) | 4.5, 8.2 | 30 (8.9) | 6.2, 13 | 12 (18) | 10, 30 |  |
| **Location** |  |  |  |  |  |  |  |  | **0.045** |
| Rural | 55 (69) | 57, 78 | 371 (54) | 50, 57 | 199 (59) | 54, 64 | 36 (55) | 42, 67 |  |
| Urban | 25 (31) | 22, 43 | 320 (46) | 43, 50 | 138 (41) | 36, 46 | 30 (45) | 33, 58 |  |
| **District** |  |  |  |  |  |  |  |  | **<0.001** |
| Balaka | 9 (11) | 5.6, 21 | 78 (11) | 9.1, 14 | 18 (5.3) | 3.3, 8.5 | 14 (21) | 12, 33 |  |
| Blantyre | 3 (3.8) | 0.97, 11 | 84 (12) | 9.9, 15 | 38 (11) | 8.2, 15 | 7 (11) | 4.7, 21 |  |
| Chikwawa | 13 (16) | 9.3, 27 | 60 (8.7) | 6.7, 11 | 16 (4.7) | 2.8, 7.8 | 5 (7.6) | 2.8, 18 |  |
| Chitipa | 12 (15) | 8.3, 25 | 81 (12) | 9.5, 14 | 27 (8.0) | 5.4, 12 | 12 (18) | 10, 30 |  |
| Kasungu | 7 (8.8) | 3.9, 18 | 57 (8.2) | 6.4, 11 | 37 (11) | 7.9, 15 | 7 (11) | 4.7, 21 |  |
| Lilongwe | 11 (14) | 7.4, 24 | 48 (6.9) | 5.2, 9.2 | 35 (10) | 7.4, 14 | 2 (3.0) | 0.53, 11 |  |
| Mzimba South | 3 (3.8) | 0.97, 11 | 62 (9.0) | 7.0, 11 | 48 (14) | 11, 19 | 7 (11) | 4.7, 21 |  |
| Phalombe | 8 (10) | 4.7, 19 | 59 (8.5) | 6.6, 11 | 37 (11) | 7.9, 15 | 4 (6.1) | 2.0, 16 |  |
| Salima | 10 (13) | 6.5, 22 | 67 (9.7) | 7.6, 12 | 52 (15) | 12, 20 | 4 (6.1) | 2.0, 16 |  |
| Thyolo | 4 (5.0) | 1.6, 13 | 95 (14) | 11, 17 | 29 (8.6) | 5.9, 12 | 4 (6.1) | 2.0, 16 |  |
| *^1^*n ();*^2^*CI = Confidence Interval; *^3^*Pearson's Chi-squared test. *Source: PPT digital acceptability Survey* | | | | | | | | | |
